# Rapid transesterification of micro-amount of lipids from microalgae via a micro-mixer reactor

**DOI:** 10.1186/s13068-015-0416-7

**Published:** 2015-12-30

**Authors:** Jiao Liu, Yadong Chu, Xupeng Cao, Yuchao Zhao, Hua Xie, Song Xue

**Affiliations:** Marine Bioengineering Group, Dalian Institute of Chemical Physics, Chinese Academy of Sciences, Dalian, 16023 China; University of Chinese Academy of Sciences, Beijing, 100049 China; Microchemical Engineering and Technology Group, Dalian Institute of Chemical Physics, Chinese Academy of Sciences, Dalian, 16023 China; National Engineering Laboratory for Methanol to Olefins, Dalian National Laboratory for Clean Energy, Dalian Institute of Chemical Physics, Chinese Academy of Sciences, Dalian, 116023 China

**Keywords:** Micro-mixer, Fatty acid composition analysis, Transesterification, Microalgae, Micro-scale sample

## Abstract

**Background:**

Fatty acid composition is an important physiological parameter of microalgae, which is taken as the third generation alternative resource of biodiesel. To boost microalgal research and applications, a convenient, rapid, and acid-catalyzed transesterification procedure that satisfies the demand for the analysis of the fatty acid composition of lipids with micro-scale samples in the high-throughput screening of microalgal strains is needed, along with the evaluation of the physiological status of microalgae in response to nutrient stress.

**Results:**

The reaction conditions of transesterification via a micro-mixer reactor were optimized as follows: 90 °C reaction temperature, 20 min reaction time, 6:1 volume ratio of H_2_SO_4_-methanol to lipid-in-hexane, and a Y-type micro-mixer with a 20-m-long extended loop that has a 0.3 mm diameter. The minimum amount of sample was decreased to 30 µg lipids. The new approach was successfully applied to the fatty acid composition analysis of soybean oil and microalgal lipids. Definitely, it could be applied to acyl related oils from different sources.

**Conclusion:**

Here, we have developed a simple and rapid method for the analysis of the fatty acid composition of lipids. The new method requires less than 20 min for transesterification and a minimum of only 30 µg lipid sample. Furthermore, a high-throughput process can be easily realized by numbering up the micro-mixer reactors. The micro-mixer reactor has great potential for applications not only in large-scale biodiesel production but also for the micro-scale analysis of microalgae fatty acid compositions.

## Background

In the past two decades, microalgae have received increasing attention due to their great potential as an alternative resource in biofuel production and in other field applications, such as pharmaceutical resources, aquaculture feeds, and human food [1]. Quantifying fatty acids (FAs) is critical in microalgal research regarding their use as biofuels, as well as in the acyl correlated field. Previous studies on microalgal screening and physiological features showed that characteristics of the changes in FAs are important indicators. For instance, Wang et al. identified C18:1n9 as a positive biomarker related to a neutral lipid content [2]. In biodiesel production, the fatty acid methyl ester (FAME) composition, greatly affects biodiesel quality, such as the cetane number, calorific value, and other indices [3, 4]. Thus, the FA composition of microalgae is a key factor when screening candidates for biodiesel production resources and during physiological studies.

Transesterification is the most common method to convert acyl into FAME or fatty acid ethyl ester, which is used to quantify or quantitate fatty acids from the source. Considering the high acid value of microalgal lipids, acid is an appropriate catalyst in transesterification for determining the FA composition of microalgal lipids, as it simultaneously catalyzes both the esterification of free FA and the transesterification of other lipids [5]. Bigelow et al. established an in situ protocol for GC–MS lipid analysis that required only 250 µg of dry sample. The transesterification was performed in tubes with caps and was catalyzed by boron trifluoride at 100 °C for 1 h [6]. We developed a direct method for the transesterification of microalgal cells that required a minimum of 300 µg of dry cells in our previous work, and the reaction time was maintained at 1 h when catalyzed with sulfuric acid at 70 °C [7]. Although the transesterification method for the analysis of the microalgal FA composition is relatively mature and only requires several hundred micrograms of samples, it is still a time-consuming and multistep process, which limits the rapid and high-throughput analysis of FA in microalgae.

The efficiency of transesterification can be increased by shortening the time to reach reaction equilibrium. The initial transesterification rate is limited by the mass transfer of the immiscible reactants in two phases. Therefore, increasing the mixing of the two immiscible reactants to accelerate the mass transfer of the transesterification system can improve the transesterification efficiency as well as the FAME yield as a result. A co-solvent has been used in transesterification to increase the solubility between reactants and thus improve the mixing and mass transfer rate between the reactants [8, 9]. Micro-reactors have the distinct ability to intensify the mass transfer of liquid–liquid two-phase reactions [10, 11]. Various types of micro-reactors used for homogeneous base-catalyzed transesterification have been investigated [12–15]. In the study by Wen et al., a 99.5 % FAME yield of soybean oil transesterification by base catalyst was achieved with only a 28 s residence time using zigzag micro-channel reactors [15]. Sun et al. developed a two-step, acid-catalyzed process of esterification followed by the transesterification of high acid value oils using a micro-structured reactor with a total reaction time of less than 15 min [16]. Except for the rapid mass transfer, the micro-scale system has several remarkable advantages compared with the batch reactor (BR): a rapid heat exchange, a high controllability, a micro-scale sample requirement, and a high throughput that is easily realized by numbering up [10, 11]. Therefore, transesterification in a micro-reactor is a promising alternative technique compared to the traditional tedious transesterification process, and it needs only a micro-sample for a rapid fatty analysis and has great potential for continuous biodiesel production.

In this work, we investigated transesterification in a micro-mixer reactor (MR) to systematically analyze FA composition. Our results demonstrate a reduction in the minimum lipid sample to 30 µg and a shortening of the overall FA composition analysis process.

## Results and discussion

### Effect of different types of MRs on transesterification

**Fig. 1:**
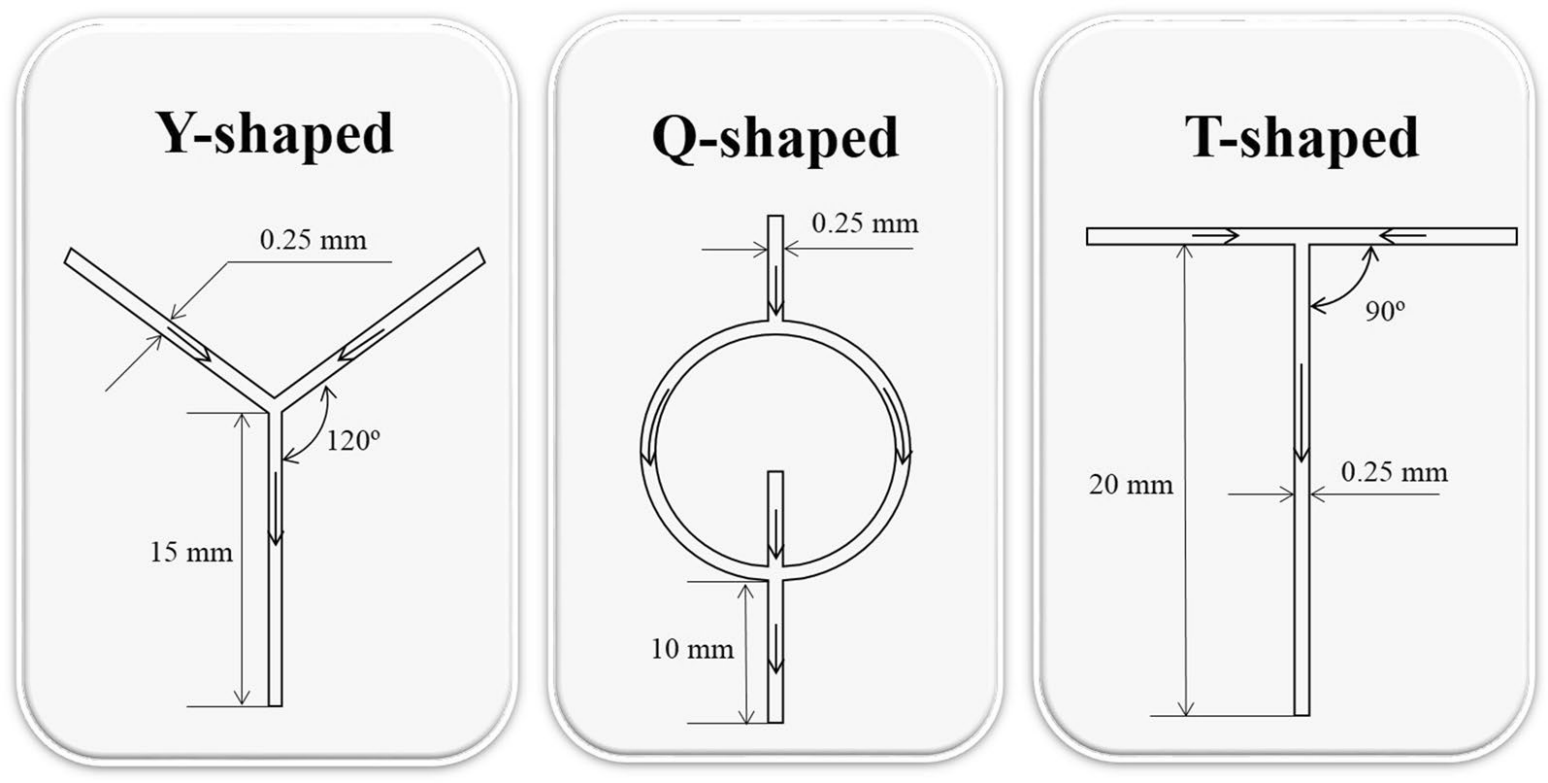
Structures of the three types of MRs

**Fig. 2:**
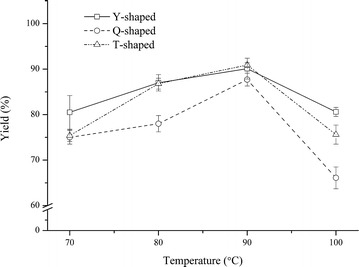
FAME yields obtained in three types of MRs at different reaction temperatures

In a MR, the mixing efficiency largely depends on geometric structures [12, 17]; temperature is also a very important reaction factor for transesterification. Here, three types of MRs with simple structures, as shown in Fig. 1, were used at different reaction temperatures to perform the transesterification of glyceryl trioleate (TAG). The diameter of the extended loop capillary was 0.3 mm, and the reaction time was 20 min at a 6:1 volume ratio of H_2_SO_4_-methanol to  lipid-in-hexane. The FAME yields obtained by transesterification in the three types of MRs are shown in Fig. 2.

For Y-typed MR, the FAME yield increased from 80.5 to 90.1 % as the temperature increased from 70 to 90 °C. However, the FAME yield decreased to 80.6 % at 100 °C. At 100 °C, the reaction color became dark, and the FA composition of the darker product, as determined by GC, was different from the product yielded under lower temperatures (data no shown). The same temperature-dependent trend was observed for the other two types of MRs as well. Previous studies have reported that the FAME yield is lower at higher reaction temperatures, and an increase in the dark color of the product has been observed [18–20]. This probably resulted from the carbonization and polymerization effect of sulfuric acid at high temperatures. Li et al. explained that this result is probably because high concentrations of sulfuric acid can burn some of the oil at high temperatures and lead to a low yield of the biodiesel product [18]. In a study by Miao et al., the FAME profiles obtained at different temperatures (100, 120, 150, and 200 °C) changed, and the yield of FAME decreased at higher temperatures [19]. Therefore, regarding the accuracy of determining FA compositions by acid-catalyzed transesterification coupled with GC, a temperature of 90 °C was the best compromise in this system.

Figure 2 illustrates the different effects of three types of MRs on the FAME yield. At 90 °C, the FAME yield was 90.1, 90.9, and 87.7 % for Y-, T-, and Q-type MRs, respectively. The higher FAME yield obtained by the Y- and T-type MRs indicated that Y- and T-type geometries could more efficiently improve mass transfer compared to the Q-type geometry in our reaction system. It has been well documented that the mixing efficiency is affected by the geometric structure of the different MR types. In work by Hsieh et al., the mixing efficiency was enhanced by increasing the mixing angle of the Y-type mixer [17]. A T-type mixer is another basic geometry design used in many studies on mixing efficiency or flow physics [21–23]. These studies demonstrated that the flow rate and MR geometry strongly affected the development of vortices, which was essential to achieve good mixing performance. To achieve higher mixing efficiency, we designed a Q-type mixer with a more complex geometry structure that may enhance the development of vortices at the intersection of the inlet flow [17, 24] compared to the T- and Y-type reactors. However, the results showed a lower mixing efficiency of the Q-type than the other two MR types, as displayed in Fig. 2. The unequal velocity of the two inlet flows and the different physical properties of the solutions [17, 24] are possible reasons for the unsuccessful design. There is no mature theoretical guidance to design an MR because there is a limited understanding of MR fluidics. Therefore, in this work, the Y-type and T-type MRs were the more efficient mixers. Considering the Y-type one is a commercial product which is more available for other researchers who wants to perform transesterification in micro-mixer reactors, the subsequent experiments were carried out using a Y-type MR at 90 °C.

### Effect of the diameter of the extended loop on transesterification in an MR

**Fig. 3:**
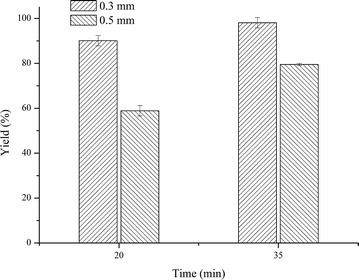
FAME yields obtained in the Y-type mixer with extended loops of different inner diameters

After mixing in the MR, the reactants began to react in the extended loop. The diameter of the extended loop, which determines the diffusion distance of the molecule and further influences the mass transfer coefficient of the whole reactor system, influences the conversion process [13, 14]. Both 20 m lengths of the extended loop with 0.3 and 0.5 mm diameters were used to investigate the effect of the diameter on transesterification by varying the total flow rate in the extended loop. Figure 3 shows the effect of the diameter of the extended loop on the FAME yield for 20 and 35 min reaction times. When the diameter was narrowed from 0.5 to 0.3 mm, the FAME yield increased from 58.9 to 90.1 % for a 20 min reaction and from 79.5 to 98.1 % for a 35 min reaction, indicating that the FAME yield increased with the decrease in the extended loop diameter. In most cases of microfluidics, a laminar flow can be expected which makes diffusion the main form of the mass transfer [24, 25]. The diffusional distance was shortened by decreasing the diameter of the extended loop, which caused a faster mass transfer during the transesterification and consequently resulted in a higher FAME yield. Similar results suggesting that shorter diameter lead to higher conversions have been reported in previous works [13, 15]. If the diameter was further reduced, a higher pressure drop would occur, which would consequently lead to increased energy consumption [26]. However, the gap between FAME yields from the 0.3 and 0.5 mm diameters were narrowed after increasing the reaction time from 20 to 35 min. Considering the increasing occurrence of blockages caused by microalgal cells in extended loops with smaller diameters, the 0.3 mm diameter extended loop was chosen for subsequent experiments regarding the transesterification of microalgal cells using MRs in our further plan.

### Optimization of transesterification in MRs

Hexane, which was chosen as the carrying reagent to bring the lipids into the MR for transesterification, considering the high viscosity and even the solid state of various types of crude lipids, was incorporated into transesterification with H_2_SO_4_-methanol system. Usually, methanol and hexane form a part of the binary immiscible liquid–liquid system. A homogeneous phase system could be obtained by changing the volume ratio of the two reagents. Therefore, the ratio of the two inlet flows determines the final fluid phase and further affects the reaction efficiency.

Before carrying out the transesterification in a MR, the effect of hexane on transesterification in a (BR) was investigated. One-sixth of the methanol volume hexane was added to reaction system and the reaction time was 30 min. The FAME yield was increased from 80.7 to 82.2 % after adding hexane which could increase oil miscibility in the mixture. The results were in agreement with previous studies that used hexane as co-solvent [9, 27]. The acceleration on transesterification makes hexane as a good carrying reagent.

**Fig. 4:**
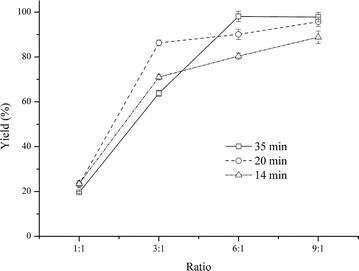
FAME yield obtained under different reaction times and ratios of the two inlet flows

The effects of the volume ratio of the two inlet flows and the reaction time on the FAME yield were investigated by varying the flow velocity. The results in Fig. 4 show that the FAME yield increased as the ratio of the two inlet flows increased. When the ratio was 1:1, the FAME yield was very low; even after prolonging the reaction time from 14 to 35 min, there was no increase in the FAME yield as expected. By increasing the ratio to 3:1, the FAME yield increased from 23.2 to 86.3 % dramatically for the 20 min reaction time. Increased FAME yields were also observed at the 14 and 35 min reaction times. The FAME yield reached 98.1 and 97.8 % when the ratio was 6:1 and 9:1 at the 35 min reaction time, respectively. A two-phase plug-flow of the immiscible solvents after pumping a 1:1 ratio of methanol and hexane into the MR and the slow mass transfer between the two-phase plug-flow resulted a low FAME yield consequently. When the ratio was increased to 6:1 and 9:1, a homogeneous phase flow was generated after pumping into the MR. Therefore, the mass transfer limitation of the two immiscible phases was significantly reduced. This result explains why the FAME yield dramatically increases as the ratio of the two phases increases. Compared with a BR, the enhancement of transesterification in the MR by adding hexane into the reaction system is more apparent. In the BR, for the 30 min reaction, the FAME yield increased from 80.7 to 82.2 % by adding hexane at one-sixth of the methanol volume. While in the MR, the FAME yield increased from 23.2 to 90.1 % for a reaction time of only 20 min by increasing the ratio from 1:1 to 6:1, as seen in Fig. 4. In addition to the ratio of the two immiscible phases, increasing the methanol-to-oil ratio was another factor that increased the FAME yield [14, 28].

It should be noted that in our study, the reaction time was changed by varying the velocity of the total inlet flow. Velocity is one of the major influencing factors of mass transfer in a micro-scale reactor [10, 17]. In the work by Dummann et al. [10], the conversion of the mass-transfer-limited reaction increased as the velocity increased at the same reaction time, which could be explained by an enhancement of the mass transfer. Usually mass-transfer-limited reactions can be improved by extending the reaction time. As shown in Fig. 4, the FAME yield was higher with the longer reaction time. When the ratio was 6:1, the FAME yield increased from 80.4 to 90.1 % by changing the reaction time from 14 to 35 min, and at the 9:1 ratio, the FAME yield increased from 88.8 to 97.8 % by increasing the reaction time from 14 to 35 min. However, when the ratio was 1:1, the FAME yield was 19.6 % at 35 min, lower than 23.5 % at 14 min, and 23.2 % at 20 min. These results demonstrated that the velocity of the fluid has a greater influence than the reaction time on the mass transfer at a low ratio of methanol and hexane, and reaction time contributes more than the velocity of the total flow to the mass transfer at a high ratio of methanol and hexane. Therefore, in order to shorten the reaction time under the premise of acceptable reaction yield, the ratio has to be optimized instead of only increasing flow velocity.

The highest yield was 98.1 % at the ratio of 6:1 for 35 min reaction time. However, the yield reached 90.1 % at 6:1 ratio for only 20 min which could guarantee the accuracy of FA analysis according to our previous work [7]. In order to meet the need of the fast FA composition analysis, the optimized condition was determined to be a 6:1 ratio for 20 min instead of 35 min reaction time.

### Minimum amount of sample required for FA composition analysis by MR

Traditionally, several milligrams or more of sample are required to perform the transesterification in a FA composition analysis procedure. Soybean oil and extracted microalgal oil of *I. zhangjiangensis* were carried out by transesterification via MR followed by GC to investigate the minimum amount of sample required. The oil was dissolved in hexane to create different concentrations: 300, 100, and 50 µg/mL. The 2 % H_2_SO_4_-methanol and oil solutions were pumped into the MR at the ratio of 6:1. The collected effluent mixture was automatically split into phases, and the upper hexane layer was analyzed by GC analysis to determine the FA compositions. According to our experience with microalgal FA composition analyses, a ± 10 % variation of each FA is acceptable [7]. Relative FA percentage with ±10 % variation was obtained based on the results from transesterification in the BR. As displayed in Table 1, compared to results of the BR, the percentages of C16:0, C18:0, C18:1n9, and C18:3n3 obtained from MR using the lipid solution at the concentration of 100 µg/mL (MR-100) and 50 µg/mL (MR-50) were not in the ±10 % variation range, and only the results of MR using 300 µg/mL solution (MR-300) met the analysis requirements. The FA compositions of *I. zhangjiangensis* are listed in Table 2, and the data that did not fall in the valid range are shown in bold fonts.

**Table 1:** FA compositions of soybean oil

FA	±10 % variation	BR	MR-300	MR-100	MR-50
C16:0	10–2.2	11.1 ± 0	11.6 ± 0.3	**12.8 ± 0.1**	**13.2 ± 0.2**
C18:0	4–4.9	4.5 ± 0.1	4.9 ± 0.4	**5.5 ± 0.1**	**6 ± 0.1**
C18:1n9	19.7–24.1	21.9 ± 0.1	23.4 ± 0.7	**24.5 ± 0.3**	**25.7 ± 0.1**
C18:1n7	1.2–1.5	1.4 ± 0	1.5 ± 0	1.5 ± 0.1	1.5 ± 0.2
C18:2n6	47.9–58.5	53.2 ± 0.1	52 ± 1	49.4 ± 0.5	47.6 ± 0.3
C18:3n3	6.5–7.9	7.2 ± 0	6.7 ± 0.5	**6.3 ± 0.1**	**6 ± 0.2**

The data shown in *bold* fonts represent those that did not fall in the valid ±10 % variation range

**Table 2:** FA compositions of extracted lipids from *Isochrysis zhangjiangensis*

FA	±10 % variation	BR	MR-300	MR-100	MR-50
C14:0	22.3–27.2	24.8 ± 0.3	24.1 ± 0.1	23.4 ± 0.3	23.3 ± 0.1
C16:0	12.8–5.7	14.3 ± 0.1	14.3 ± 0.2	14.6 ± 0.2	15.1 ± 0.2
C16:1n7	8.4–10.3	9.3 ± 0	9.4 ± 0.1	9.1 ± 0	9.1 ± 0.2
C16:2n4	0.7–0.9	0.8 ± 0	0.8 ± 0.1	0.8 ± 0	0.8 ± 0
C18:0	1–1.3	1.2 ± 0	1.3 ± 0	**1.8 ± 0.2**	**2.2 ± 0.1**
C18:1n9	12.1–14.7	13.4 ± 0.1	14 ± 0.6	14.9 ± 0.2	**15.6 ± 0.3**
C18:1n7	1.1–1.4	1.2 ± 0.1	1.4 ± 0	1.4 ± 0	1.4 ± 0
C18:2n6	8.4–10.2	9.3 ± 0.1	9.4 ± 0.2	9.7 ± 0.1	9.9 ± 0.1
C18:3n3	9.9–12.1	11 ± 0.2	10.6 ± 0.2	10.4 ± 0.2	10 ± 0.1
C18:4n3	8–9.8	8.9 ± 0.1	8.6 ± 0.1	8.3 ± 0	**7.6 ± 0.1**
C18:5n3	1.8–2.2	2 ± 0.3	2.2 ± 0.3	2.1 ± 0.1	1.9 ± 0.2
C22:6n3	3.4–4.2	3.8 ± 0.1	3.9 ± 0.9	3.4 ± 0.1	**3.1 ± 0.1**

The data shown in *bold* fonts represent those that did not fall in the valid ±10 % variation range

In our previous work [7], the minimum amount of sample to accurately perform an FA composition analysis in BR was 300 µg of dry microalgal cells. When the sample was lower than 300 µg, the relative percentage of FA dramatically varied. We presumed it was the different dissolving capacities of FAME with various chain-length and unsaturation degrees in hexane and methanol that led to the change of FAME composition determined when using less than 300 µg of dry cell sample by transesterification. When the total amount of FA is to be several micrograms, the residues remaining in the water phase account for a proportion that cannot be neglected for each FA after the distribution equilibrium between the two phases. When the amount of the sample is large enough, the residues remaining in the water phase account for a small portion of the total FA and can be neglected, making little difference to the overall FA composition. Therefore, there should be a minimum amount of sample required for credible FA composition analysis by transesterification. From what has been discussed above, the concentration 300 µg/mL lipid solution was proved to be minimum concentration for accuracy FA composition analysis via MR. In addition, in the MR system, 100 µL lipid solution is enough for one run of transesterification, which means only 30 µg lipid (concentration 300 µg/mL, 100 µL) is needed to perform a credible FA composition analysis.

To verify the accuracy and universality of the transesterification method via MR using only 30 µg microalgal lipids, FA composition analysis of three more species of microalgae were performed. Relative FA percentage with ±10 % variation was obtained based on the results from transesterification in the BR in the same way with soybean oil and lipids extracted from *I. zhangjiangensis*. The results of ±10 % variations and the measured FA compositions in MR using 30 µg microalgal lipids (100 µL of lipid solutions at 300 µg/mL concentration) and BR were list in Table 3. Compared to the FA compositions obtained from BR, the results obtained from MR using only 30 µg lipids of the three species of microalgae were all in the ±10 % variations. Therefore, the method of FA composition analysis via MR using only 30 µg microalgal lipids is reliable and can be applied to other microalgae.

**Table 3:** FA compositions of extracted lipids from three species of microalgae

FA	*Chaetoceros* sp.			*Chlorella Vulgaris*			*Nannochloropsis*		
	±10 % variation	BR	MR-300	±10 % variation	BR	MR-300	±10 % variation	BR	MR-300
C14:0	32.2–39.4	35.8 ± 0.1	35.7 ± 0.2	1.4–1.7	1.5 ± 0.2	1.9 ± 0.3	5.3–6.4	5.9 ± 0.1	6 ± 0
C16:0	10.2–12.5	11.3 ± 0	11.8 ± 0	35.8–43.7	39.8 ± 0.2	39.9 ± 0.2	24.6–30	27.3 ± 0.1	28 ± 0.7
C16:1n7	22.7–27.8	25.2 ± 0.2	25.1 ± 0.1				21.5–26.2	23.8 ± 0.2	23.9 ± 0.1
C16:2n4	6.7–8.2	7.4 ± 0.1	7.3 ± 0						
C16:3n4	5.6–6.8	6.2 ± 0	6.1 ± 0.1	4–4.8	4.4 ± 0	4.4 ± 0.1			
C18:0	1.2–1.5	1.3 ± 0	1.5 ± 0	8.6–10.5	9.5 ± 0.1	9.8 ± 0.1	1.1–1.4	1.3 ± 0.1	1.5 ± 0.1
C18:1n9	0.9–1.1	1 ± 0.1	1.3 ± 0.1	19–23.2	21.1 ± 0.1	20.8 ± 0.1	3.9–4.8	4.3 ± 0	4.8 ± 0.2
C18:1n7	1.1–1.4	1.2 ± 0.1	1.3 ± 0	1.3–1.6	1.5 ± 0.1	1.5 ± 0.1	0	0	0
C18:2n6	0.8–1	0.9 ± 0	0.9 ± 0	13.4–16.3	14.8 ± 0.1	14.4 ± 0.1	2–2.5	2.2 ± 0	2.4 ± 0
C18:3n3				6.7–8.1	7.4 ± 0	7.2 ± 0.1			
C18:5n3									
C20:4n6	2–2.5	2.3 ± 0	2.2 ± 0				3.8–4.6	4.2 ± 0.2	4.2 ± 0.1
C20:5n3	6.5–7.9	7.2 ± 0.1	6.7 ± 0				27.9–34.1	31 ± 0.2	29.3 ± 0.9

## Conclusions

MRs have been proven to enhance the mixing efficiency of the transesterification reactants and therefore greatly shorten the reaction time. Here, we have developed a simple and rapid method for a FA assay of crude lipids. The time of transesterification of lipids is only 20 min that much shorter than the conventional condition, and the minimum sample required is 30 µg lipid for the micro-scale system for the analysis of FA composition. Furthermore, a high throughput process can be easily realized by numbering up the MRs, which will greatly influence the rapid FA composition analysis of microalgal screening and determining microalgal physiological status during cultivation. The micro-reactor has great potential for many applications in both large-scale production of biodiesel or rapid FA composition analysis of micro-scale microalgae samples. For future studies, we will expand the applicability of this method to various forms of samples, such as the direct transesterification of fresh microalgae culture solution without further treatment.

## Methods

### Materials

Chromatographic grade n-hexane and methanol were purchased from J&K Scientific Ltd (China). Sulfuric acid (purity 95–98 %) was analytical grade and was obtained from Tianjin Kemiou Chemical Reagent Co., Ltd (China). Chemically pure glyceryl trioleate (TAG) was purchased from Sinopharm Chemical Reagent Co., Ltd (SCRC) China. Methyl hexadecanoate was purchased from Sigma-Aldrich (USA). Soybean oil was purchased from a local supermarket, and the microalgal lipids were prepared using *I. zhangjiangensis* and *Chaetoceros* sp. cultured in our lab. *Chlorella Vulgaris* and *Nannochloropsis* were obtained from South China University of Technology and Beihang University, respectively.

Syringe pumps were purchased from Baoding Longer Precision Pump Co., Ltd. The Y-shaped MR is a simple triple valves with a 0.25 mm inner diameter from Dalian Elite Analytical Instruments Co., Ltd. The T-shaped and Q-shaped with 0.25 mm inner diameter MRs used in the studies were manufactured using glasses.

### Process of transesterification via MR

Certain concentration of H_2_SO_4_-methanol (v/v H_2_SO_4_/methanol) and the oil samples dissolved in hexane were fed by two syringe pumps into the MR from two entrances at specified speeds and ratios. It should be noted that the ‘ratio’ in this article refers to the ratio of the H_2_SO_4_-methanol to oil-hexane volume pumped into the inlets of the MR. The extended loop connected to the output of the MR was a 20-m-long PTFE capillary (diameters = 0.3, 0.5 mm) that was immersed in the constant temperature oil bath for temperature control. The extended capillary tube and the collection bottle were immersed in an ice-water bath to cool the mixture. The reaction time was changed by varying the velocity of the total velocity of the two inlet flow. For example, when the reaction time was 20 min, the actual flow velocities of the two inlets were 60 µL/min and 10 µL/min using the 20 m extended capillary tube with 0.3 mm diameter. The collection bottle was loaded with enough distilled water beforehand to stop the reaction and make the effluent mixture automatically separate. The mixture automatically formed two phases, and the upper hexane layer containing the FAMEs was analyzed by GC to determine FA compositions and the FAME yield according to our previous work [7]. The FAME yield was calculated using the following equation:

1 $${\text{FAME yield }}\left( \% \right) \, = \, \left( {\text{FAME mass}} \right)/\left({\text{oil mass}} \right) \, \times {100} \;\%$$

The measurements of the values used in the tables and figures were performed in triplicate during the whole experiment.

## Abbreviations

FA: fatty acid
MR: micro-mixer reactor
FAME: fatty acid methyl ester
TAG: glyceryl trioleate
GC: gas chromatography
BR: batch reactor
MR-50, MR-100, MR-300: transesterification in MR using lipid solution at concentration of 50 µg/mL, 100 µg/mL, 300 µg/mL, respectively
